# Ambient Solar Radiation Predicts Psoriasis Treatment Intensity

**DOI:** 10.1111/phpp.70014

**Published:** 2025-03-28

**Authors:** Brad R. Woodie, Alan B. Fleischer

**Affiliations:** ^1^ University of Cincinnati College of Medicine Cincinnati Ohio USA; ^2^ Department of Dermatology University of Cincinnati College of Medicine Cincinnati Ohio USA

**Keywords:** *All of Us*, biologic, immunosuppression, psoriasis, sunlight, ultraviolet

## 1 Introduction

Observations of seasonal variation in psoriasis severity (worsening in winter and improving in summer) suggest a beneficial effect of ambient solar radiation [1, 2]. Additionally, “climate therapy” using intentional sunlight exposure reduces psoriasis severity [3]. Interestingly, an analysis of psoriasis clinical trial participants by Okun and Okun [4] found no relationship between psoriasis severity and the ambient ultraviolet radiation at investigator sites. Also, Jacobson et al. [5] compared the psoriasis prevalence in extant studies by the mean country latitude and found no substantial correlation. Using a diverse sample of the United States, our study assessed the association of annual local solar radiation with psoriasis treatment intensity.

## 2 Methods

We analyzed de‐identified participant data from the National Institutes of Health's *All of Us* Research Program version 7, which includes data from 2017 to 2022. Seven thousand seven hundred forty eight participants with a diagnosis of psoriasis were identified. To reduce the likelihood of patients requiring systemic immunosuppressive treatments for other conditions, we excluded participants with diagnoses of psoriatic arthritis, rheumatoid arthritis, systemic lupus erythematosus, atopic dermatitis, uveitis, Crohn's disease, and ulcerative colitis. Our final cohort included 5179 participants after excluding participants with missing values for 3‐digit ZIP code or body mass index (BMI) as obtained from the participant's electronic health record. This study followed the Strengthening the Reporting of Observational Studies in Epidemiology (STROBE) reporting guidelines.

Treatment intensity was assessed by the presence or absence of a systemic psoriasis treatment or phototherapy (Table S1). Mean annual global horizontal irradiance (GHI) data (1998–2016) for the contiguous United States by ZIP code was obtained from the National Renewable Energy Laboratory [6]. Deprivation index (a composite score calculated by the *All of Us* Program using measures of poverty, education, housing, and lack of health insurance at the census tract level) was included to control for confounding related to differences in access to care.

Descriptive statistics of mean and standard deviation or frequency and percent were calculated. GHI was divided into quartiles: under 3.97 kWh/m^2^/day (Quartile 1, e.g. Seattle, Washington), 3.97 to 4.05 (Quartile 2, e.g. Chicago, Illinois), 4.05 to 4.92 (Quartile 3, e.g. Atlanta, Georgia), and over 4.92 (Quartile 4, e.g. Los Angeles, California). Multivariable logistic regression compared treatment odds by participant ZIP code GHI, adjusting for deprivation index, age, BMI, sex assigned at birth (hereafter “sex”), race, and ethnicity. Significance was set at two‐tailed *p* < 0.05, and analysis was performed using SAS Studio in the *All of Us* Researcher Workbench.

## 3 Results

**FIGURE 1:**
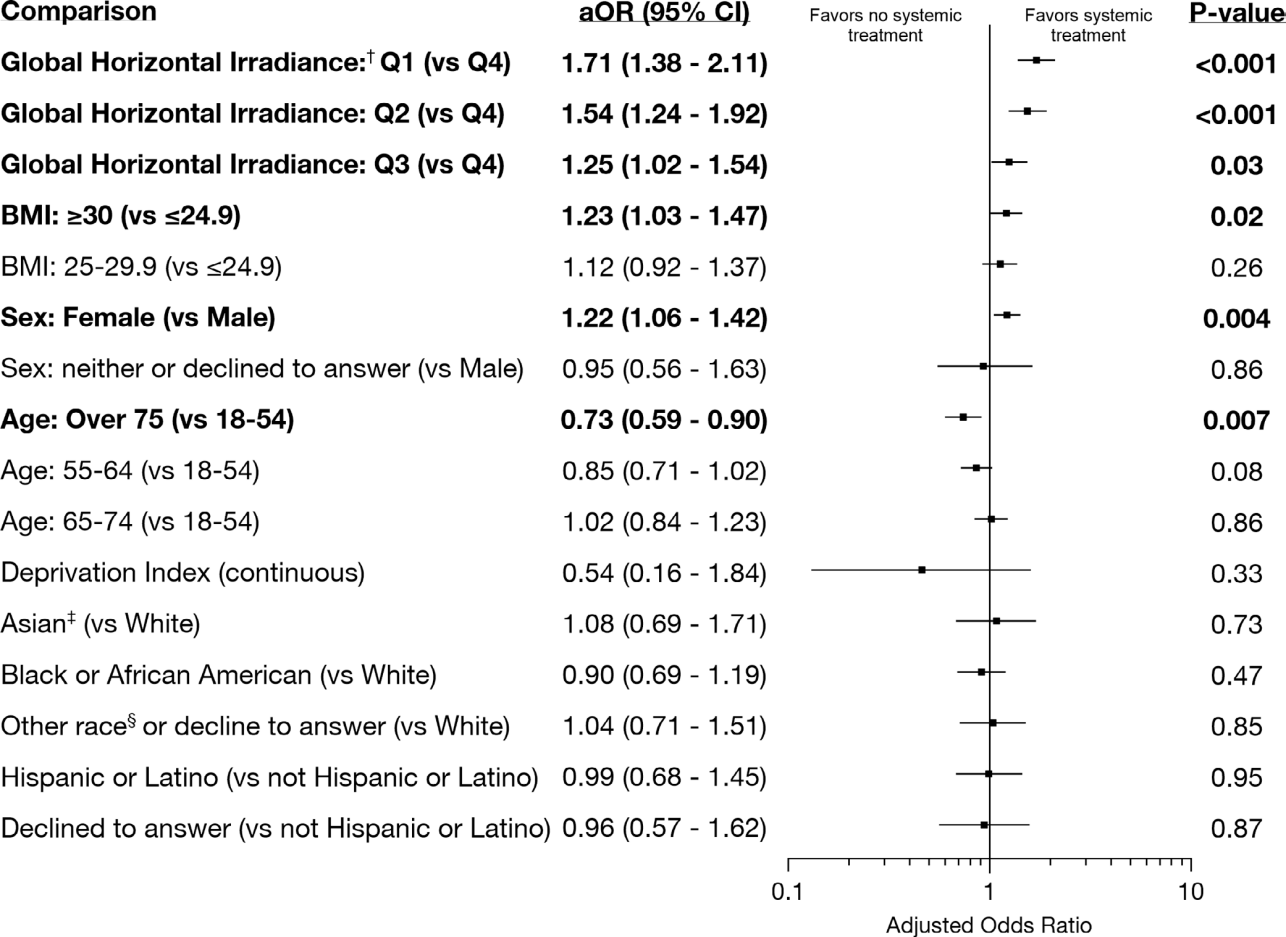
Multivariable logistic regression for the odds of receiving a psoriasis systemic therapy or phototherapy. ^†^Quartile 1 (Q1): Global horizontal irradiance (GHI) under 3.97 kWh/m^2^/day (e.g., Seattle, Washington). Q2: GHI of 3.97 to 4.05 (e.g., Chicago, Illinois). Q3: 4.05 to 4.92 (e.g., Atlanta, Georgia). Q4: Over 4.92 (e.g., Los Angeles, California). ^‡^Race and ethnicity were obtained from self‐report and were included in this analysis as a goal of *All of Us* to collect and report data from a diverse group of participants. ^§^“Other” races included American Indian or Alaskan Native, Middle Eastern or North African, Native Hawaiian or Other Pacific Islander, and more than one population.

Of the 5179 participants with psoriasis in the study, 1028 received systemic treatment or phototherapy (Table 1). Lower GHI of the participants' ZIP code was associated with higher odds of receiving systemic treatment or phototherapy (Figure 1). Obesity and female sex were also associated with higher odds of systemic treatment or phototherapy. Age over 75 was associated with lower odds of systemic therapy or phototherapy. We did not find an association between deprivation index, race, or ethnicity and treatment intensity.

**TABLE 1:** Characteristics of patients in the sample by systemic psoriasis therapy status.

Characteristic	No systemic treatment or phototherapy (*N* = 4151), No. (%)	Systemic treatment or phototherapy (*N* = 1028), No. (%)
Deprivation Index, mean (SD)	0.31 (0.06)	0.30 (0.05)
Global horizontal irradiance:^a^ Q1	976 (75.9)	310 (24.1)
Global horizontal irradiance: Q2	1179 (79.2)	309 (21.8)
Global horizontal irradiance: Q3	887 (81.0)	208 (19.0)
Global horizontal irradiance: Q4	1109 (84.7)	201 (15.3)
Age: 18–54	1212 (78.4)	334 (21.6)
Age: 55–54	877 (78.4)	242 (21.6)
Age: 65–74	1200 (81.0)	281 (19.0)
Age: > 75	862 (83.4)	171 (16.6)
BMI: ≤ 24.9 kg/m^2^	961 (82.0)	211 (18.0)
BMI: 25–29.9 kg/m^2^	1274 (80.7)	304 (19.3)
BMI: ≥ 30 kg/m^2^	1916 (78.9)	513 (21.1)
Female sex	2299 (78.5)	629 (21.5)
Male sex	1752 (82.2)	379 (17.8)
Other sex or decline to answer	100 (83.3)	20 (16.7)
Race^b^: Asian	96 (79.3)	25 (20.7)
Race: Black or African American	308 (80.6)	74 (19.4)
Race: Other^c^ or decline to answer	741 (80.6)	178 (19.4)
Race: White	3006 (80.0)	751 (20.0)
Ethnicity: Hispanic or Latino	545 (80.4)	133 (19.6)
Ethnicity: Not Hispanic or Latino	3424 (80.0)	854 (20.0)
Ethnicity: Decline to answer	182 (81.6)	41 (18.4)

^a^ Quartile 1 (Q1): Global horizontal irradiance (GHI) under 3.97 kWh/m^2^/day (e.g., Seattle, Washington). Q2: GHI of 3.97 to 4.05 (e.g., Chicago, Illinois). Q3: 4.05 to 4.92 (e.g., Atlanta, Georgia). Q4: over 4.92 (e.g., Los Angeles, California).

^b^ Race and ethnicity were obtained from self‐report and were included in this analysis as a goal of *All of Us* to collect and report data from a diverse group of participants.

^c^ “Other” race included American Indian or Alaskan Native, Middle Eastern or North African, Native Hawaiian or Other Pacific Islander, and more than one population.

## 4 Discussion

Our study shows that lower levels of ambient solar radiation are associated with higher odds of receiving systemic therapy or phototherapy for psoriasis, with these treatments serving as possible indicators of more severe disease. The lack of association between severity and ultraviolet radiation seen by Okun and Okun [4] may have been due to clinical trial participants having stable moderate‐to‐severe psoriasis and being instructed to limit their environmental ultraviolet radiation exposure. Of note, the study by Jacobson et al. [5] of latitude and psoriasis prevalence excluded the United States and China due to the range of latitudes spanned by these countries. Solar irradiance also varies with factors beyond latitude, such as altitude and cloud coverage [6].

The associations of obesity, sex, and age with treatment seen in our study align with prior research. Obesity is associated with psoriasis severity, and weight loss leads to a reduction in the Psoriasis Area and Severity Index (PASI) [7]. Although male sex is associated with higher PASI scores, female sex is associated with a greater likelihood of receiving biologic treatment [8]. Increasing age is associated with a lower intensity of psoriasis treatment [9]. No significant association between the deprivation index and treatment status is encouraging from a health equity perspective.

Limitations include the *All of Us* Program being a volunteer‐based sample of only the United States. Also, we cannot account for people's movement to areas of different solar irradiance or outdoor activities that increase ultraviolet radiation exposure. Clothing coverage is another determinant of personal solar radiation exposure [10] for which we could not account. Nonetheless, we add to the literature on environmental components of psoriasis by suggesting that lower ambient solar radiation is associated with more frequent systemic psoriasis treatment.

## Author Contributions

B.R.W. and A.B.F. authors were involved in conceptualization, methodology, formal analysis, writing, and editing. B.R.W. performed the data curation and prepared the original draft.

## Ethics Statement

No ethical approval was needed for this study because participant data are deidentified.

## Conflicts of Interest

A.B.F. is a consultant for Bluefin and Incyte (fees). He is an investigator for Amgen, Bayer, Biogen, CellDex, Galderma, Incyte, Leo, and UCB (research support). He is a speaker for Imedic Healthcare Solutions (Hyderabad India). B.R.W. declares no conflicts of interest.

## Supporting information


Table S1

## Data Availability

This study used the *All of Us* Research Program's Controlled Tier Dataset version 7, available to authorized users on the Researcher Workbench. Global horizontal irradiance data were obtained from the National Renewable Energy Laboratory at https://www.nrel.gov/gis/solar‐resource‐maps.html.
