# Supplementary material for: Ambient Solar Radiation Predicts Psoriasis Treatment Intensity
Source: Photodermatol Photoimmunol Photomed. 2025 Mar 28;41(3):e70014. doi: 10.1111/phpp.70014 (PMC11953459; doi:10.1111/phpp.70014)
Supplement: Supplementary file 1 — Table S1 [file PHPP-41-e70014-s001.docx]

**Supplementary Digital Content**

**Supplemental Table 1: The number of patients receiving systemic psoriasis therapy or phototherapy.**

| **Name** | **Frequency ^a^** | **Percent** |
| --- | --- | --- |
| **Acitretin** | 0 | 0 |
| **Adalimumab** | 138 | 13.4 |
| **Apremilast** | 156 | 15.2 |
| **Brodalumab** | 5 | 0.5 |
| **Certolizumab pegol** | 4 | 0.4 |
| **Cyclosporine** | 158 | 15.4 |
| **Etanercept** | 52 | 5.1 |
| **Guselkumab** | 42 | 4.1 |
| **Infliximab** | 19 | 1.9 |
| **Ixekizumab** | 36 | 3.5 |
| **Methotrexate** | 230 | 22.4 |
| **Risankizumab** | 32 | 3.1 |
| **Secukinumab** | 85 | 8.3 |
| **Tildrakizumab** | 3 | 0.3 |
| **Ustekinumab** | 67 | 6.5 |
| **Phototherapy ^b^** | 4 | 0.6 |

^a^ Frequencies represent only the most recently prescribed systemic medication per participant.

^b^ Four patients received phototherapy, three of whom also received another systemic medication.
